# Impact of Fireworks Industry Safety Measures and Prevention Management System on Human Error Mitigation Using a Machine Learning Approach

**DOI:** 10.3390/s23094365

**Published:** 2023-04-28

**Authors:** Indumathi Nallathambi, Padmaja Savaram, Sudhakar Sengan, Meshal Alharbi, Samah Alshathri, Mohit Bajaj, Moustafa H. Aly, Walid El-Shafai

**Affiliations:** 1 Department of Computer Applications, SRM Institute of Science and Technology, Ramapuram Campus, Chennai 600089, India; 2 Research Scholar, Department of Computer Science and Engineering, Kalasalingam Academy of Research and Education, Virudhunagar 626124, India; 3 Department of Computer Science and Engineering, Keshav Memorial Institute of Technology, Hyderabad 500029, India; 4 Department of Computer Science and Engineering, PSN College of Engineering and Technology, Tirunelveli 627152, India; 5 Department of Computer Science, College of Computer Engineering and Sciences, Prince Sattam bin Abdulaziz University, Alkharj 11942, Saudi Arabia; 6 Department of Information Technology, College of Computer and Information Sciences, Princess Nourah bint Abdulrahman University, P.O. Box 84428, Riyadh 11671, Saudi Arabia; 7 Department of Electrical Engineering, Graphic Era (Deemed to be University), Dehradun 248002, India; 8 Graphic Era Hill University, Dehradun 248002, India; 9 Applied Science Research Center, Applied Science Private University, Amman 11937, Jordan; 10 Electronics and Communications Engineering Department, College of Engineering and Technology, Arab Academy for Science, Technology and Maritime Transport, Alexandria 51718, Egypt; 11 Security Engineering Lab, Computer Science Department, Prince Sultan University, Riyadh 11586, Saudi Arabia; 12 Department of Electronics and Electrical Communications Engineering, Faculty of Electronic Engineering, Menoufia University, Menouf 32952, Egypt

**Keywords:** fireworks industry, human factors, machine learning, environmental sustainability, mitigation, human disaster

## Abstract

In the fireworks industry (FI), many accidents and explosions frequently happen due to human error (HE). Human factors (HFs) always play a dynamic role in the incidence of accidents in workplace environments. Preventing HE is a main challenge for safety and precautions in the FI. Clarifying the relationship between HFs can help in identifying the correlation between unsafe behaviors and influential factors in hazardous chemical warehouse accidents. This paper aims to investigate the impact of HFs that contribute to HE, which has caused FI disasters, explosions, and incidents in the past. This paper investigates why and how HEs contribute to the most severe accidents that occur while storing and using hazardous chemicals. The impact of fireworks and match industry disasters has motivated the planning of mitigation in this proposal. This analysis used machine learning (ML) and recommends an expert system (ES). There were many significant correlations between individual behaviors and the chance of HE to occur. This paper proposes an ML-based prediction model for fireworks and match work industries in Sivakasi, Tamil Nadu. For this study analysis, the questionnaire responses are reviewed for accuracy and coded from 500 participants from the fireworks and match industries in Tamil Nadu who were chosen to fill out a questionnaire. The Chief Inspectorate of Factories in Chennai and the Training Centre for Industrial Safety and Health in Sivakasi, Tamil Nadu, India, significantly contributed to the collection of accident datasets for the FI in Tamil Nadu, India. The data are analyzed and presented in the following categories based on this study’s objectives: the effect of physical, psychological, and organizational factors. The output implemented by comparing ML models, support vector machine (SVM), random forest (RF), and Naïve Bayes (NB) accuracy is 86.45%, 91.6%, and 92.1%, respectively. Extreme Gradient Boosting (XGBoost) has the optimal classification accuracy of 94.41% of ML models. This research aims to create a new ES to mitigate HE risks in the fireworks and match work industries. The proposed ES reduces HE risk and improves workplace safety in unsafe, uncertain workplaces. Proper safety management systems (SMS) can prevent deaths and injuries such as fires and explosions.

## 1. Introduction

Natural and manufactured disasters affect millions of individuals worldwide yearly [1]. Human lives are frequently lost as a direct consequence of these occurrences. Disasters not only result in human deaths but also have a significant detrimental effect on property and the environment. Operations in disaster management consider the times before, during, and after a disaster event to mitigate its impacts on the economy, safeguard lives and property, restore order, and restore normality to the damaged area [2]. Information technology (IT) and Artificial Intelligence (AI), in general, can significantly aid in making sound decisions in the terms of the physical failure and improvement of operational disaster processes and the magnitude of the disasters. In recent decades, machine learning (ML) and deep learning (DL) developments have been pivotal for more effective and informed disaster risk management [3], which is crucial in reducing the magnitude and consequences of disasters. For example, there is a high risk of disastrous death/severe injury to workers, property, and the environment if an accident occurs at a fireworks storage system or manufacturing company. Researchers have proposed many studies over the years to achieve a better understanding of the causative factors of these accidental deaths. More recently, the beneficial effects of the safety management system (SMS) have been examined. Accident frequency and severity make the fireworks industry (FI) among the poorest [4].

A growing nation needs industrial development. In India, fireworks play a crucial role in industrial development. People in and around Sivakasi, Tamil Nadu, benefit from many job opportunities [5]. In high-risk sectors, secured data collection and analysis are key components of the SMS. Human factor (HF) specialists are mainly involved in identifying occurrences caused by HE [6]. India’s “fireworks capital” is Tamil Nadu’s Sivakasi. India’s second-largest fireworks manufacturer, the town, makes 90% of India’s fireworks. Sivakasi’s 700 industry produces USD 360 million worth of fireworks annually. Sivakasi is known for its firecrackers, matchbox, and printing industries. These industries employ over 25,000 people and generate approximately USD 20 billion [7]. Hazardous chemicals are used in different manufacturing processes at this company.

Explosions occur because of this. Preventing fatalities and serious injuries is the priority for a SMS [8]. Accidents, near misses, injuries, fatalities, and explosions happen in this industry because environmental conditions react with chemical substances, static electricity, friction, and HE. “*Safety*” refers to freedom from danger, accident, risk, and unique safety features. According to the Factories Act, employee protection is paramount. Employees are at risk from fencing machinery, dust, or working without safety measures when handling raw materials. Employees will work peacefully if the factory takes safety measures.

Humans experience sensitive, strong feedback when exposed to or interacting with a hazard. The pressure of different roles in the workplace can limit employee ability to develop trusted relationships, which is required for sustaining a high level of motivation and developing efficient company and SMS protocols. Reporting incidents and accidents as well as the ensuing improvements should foster knowledge and understanding of the workplace to initiate substantial HF and SMS protocol additions. The effectiveness of incident and accident reporting may be improved by implementing a new perspective on both. A SMS is improved by addressing HFs and reviewing human interactions and the risks associated with communication and emotions. Burns and choking caused by fires and explosions are the primary causes of accidental death in fireworks accidents.

Additionally, FI accidents cost the public money, and most people still do not know enough about how safe fireworks are. Operational HEs are fires/explosions that happen by accident during production, handling, or in the warehouse due to carelessness on the part of workers. Fires at pyrotechnic plants have also been caused by accidents that happened because of poor pyrotechnics. The main goals of safety and health at work are to protect workers from hazards in the workplace and give them more ways to reduce risks. 

Any Fire Cracker Explosives Manufacturer (FCEM) that is SMS covered must follow the standard specifications, and the basic procedures are highly relevant considering the risks associated with FCEMs: an SMS is critical to FCEM infrastructures. The proper SMS can prevent deaths and injuries such as fires and explosions. Regulation according to the SMS standard will significantly contribute to the safety of rescue personnel, members of the public, workers, and manufacturers. It is also in the best interests of businesses to ensure their employees are responsible for compliance, as this will reduce the risk of injury and causing property damage. In the presence of sufficient initiating energy, explosives are chemical substances, mixtures, or devices that will detonate so there need to be procedures for safety. Preventive measures must be applied when handling explosives. During manufacturing, those who manage and use explosive materials must prevent accidental exposure to source materials of initiating energy [9].

(A) *Accidents:* Accidents caused by a device that burns explosive or flammable substances are possible when the user fails to follow the directions and safety measures. Misclassification and mislabeling of the explosive material in the fireworks were discovered. Accidents caused by unsafe conditions and unsafe acts by workers can also be greatly reduced. Careless handling, impact loading, improper stacking, and dragging are some of the unsafe acts and human errors that occur during the transportation of manufactured goods and result in accidents. Friction, careless errors, automatic failures, power leaks, and lightning effects are some causes of accidents.
(B) *Incidents:* Loss-causing events are not included in the definition of an accident. Chance alone prevents an incident from becoming an accident, and near misses or near hits are also important factors in accident damage or injury severity. Fire incidents are likely to happen during the dry season.

Reporting incidents and accidents should help people understand the work environment and lead to improvements in HFs and safety culture. However, with the demand for fireworks going up, a plant may cut back on safety measures to speed up production, leading to more accidents. Human suffering, lost productivity, and exorbitant health care expenses are all consequences of these incidents.

(C) *Process Safety Information (PSI):* For all PSI-covered mechanisms, employers must summarize written PSI relating to highly hazardous chemicals and process equipment. All other components of PSI were laid into action without PSI. PSI is written to aid in identifying and understanding process hazards by the employer and employees operating the process [10]. Information technology, the process, information on the apparatus used, and the risks associated with the highly hazardous resources used or produced by the process must be included in the PSI.
(D) *Process Hazards Analysis (PHA):* When dealing with hazardous chemicals, it is essential to conduct a method hazard assessment to correctly identify and assess any potential risks that may arise. Personnel familiar with manufacturing and engineering operational processes, at least one person acquainted with the procedure being tested, and at least one person knowledgeable in the specific process hazard analysis methods used are all considered necessary in a PHA group. To determine what causes fires and explosions, the team examines the conditions that often precede them. To effectively control identified hazards or lessen their effects, the PHA team must suggest additional safeguards. A passive or natural SMS of hazard control and the proposal of other SMSs of work and standard operating procedures are all case studies of safety precautions [11].
(E) *Operating Procedures (OP):* Detailed rules on how to carry out the multiple tasks associated with each OP in a manner that is safe and compliant with the PSI must be evidenced and made available to workers. The steps for normal operations, and those for anxious conditions, temporary functions, start-up, and a case of emergency delay, must be stated in OP in clear and concise terms. OP must also cover significant PSI, such as the real risks encountered during the process. One way to reduce the number of people in physical danger is to ensure that only authorized personnel are lawfully permitted in potentially dangerous places.
(F) *Training:* Each worker who operates a process supported by the SMS must receive initial and ongoing training from their employer. The topics of OP, safe work practices, and automatic shutdown procedures are discussed in training, in addition to the hazards to health and safety specific to each process. Each employee may have a unique level of activity. Workers in the explosives and pyrotechnics manufacturing industries, including those in maintenance and contractor roles, must thoroughly understand the health and safety risks posed by the materials and processes they use to take adequate precautions. The standard operating procedures, design parameters, care and maintenance of equipment, and emergency procedures, such as methods for determining the presence or release of toxic materials in the workplace, emergency warning signals, and how to proceed if the alert is activated, are all discussed in detail during training [12].
(G) *Mechanical Integrity (MI):* To ensure the MI of all critical process components, the FCEM must implement rigorous and systematic procedures. Fire extinguishers, storage areas, relief and venting systems, emergency shutdown systems, and controls must all be part of an employer-mandated MI program. In addition, employers should include all devices that interface with explosive material in the MI program, not just those required by the PSM benchmark, according to the FCEM.

An accident is an unplanned, undesirable event that causes loss.

The preceding definition of an accident excludes events that could have resulted in the loss. These situations are frequently called incidents, near failures, close calls, or near hits. To determine a connection between accidents and incidents, many research studies have been performed. Icebergs and triangles demonstrate these instances [13].

Basic Accident or Incident Causation accident investigations can always teach important lessons. However, our laws require employers to be proactive, spot hazards, and help make sure they are appropriately regulated. *“An accident waiting to happen*” is the phrase used to describe any risk that is not sufficiently controlled. That which poses a risk of injury is called a hazard.

Hazards are divided into different types:

- Physical: Mechanical devices, as well as electrical, thermal, acoustic, gravitational, and highly flammable raw materials.
- Chemical: Toxic gases and materials such as asbestos, sulfuric acid, and carbon monoxide.
- Biological: Influenza A, Hepatitis C, and Legionnaires’ Disease.
- Psychological: A state of shock and anxiety.

Uncontrolled/controlled hazards almost always result from underlying or organizational failures and produce unsafe acts or conditions. During an inspection, unsafe conditions such as trailing cables, oil spills on floors, missing guards, and unsecured ladders are typically easy to spot. During random inspections, it is more challenging to identify dangers such as putting people on a forklift, machining processes without safety glasses, or climbing storage racks. As a result of the fact that many unsafe acts lead to unsafe conditions, it is crucial to try and identify the source of each unsafe condition. Failure at the core of the problem almost always leads to unsafe acts and environments. Poor planning, unclear responsibilities, and inadequate supervision are just a few examples of what can go wrong with insufficient information or training. Many accidents are traced back to a failure of management control, and these underlying failures have common symptoms.

*Predefined Condition:* An injury can be caused in many ways by a FI that is not functioning correctly. For instance, if the FCEM is misused, it may explode too soon, before users have achieved a safe zone. Finally, fireworks such as bottle rockets that are designed to skyrocket through the air have the potential to take unpredictable flight paths, which could result in people being injured, such as onlookers, or damage to nearby vehicles and buildings. Additionally, a bad fuse could set off the explosive powders in the fireworks in a way the maker did not plan, causing the fireworks to go off in a way they did not expect. Fireworks injuries can be severe, resulting from improper use or a manufacturing flaw. Fireworks can cause minor stings, third-degree burns, and loss of eyesight from sparks and debris [14].

*Safety of the FI:* The principal risks associated with the FI are toxic release, fire, and explosion. There is a high potential for injury to people and damage to property due to fire. Static electricity is among the standard types of hazards in the FI. Many fireworks also include perchlorate oxidizers, which help create the oxygen required for explosions. River systems, reservoirs, and drinkable water may become heavily polluted when these disintegrate in water. In conclusion, fireworks produce a fine cloud of smoke and dust particles that pollute the air. When handling chemicals that can explode, one should always use the proper Personal Protective Equipment (PPE), such as protective clothing, a lab coat or apron, safety goggles worn with a face shield, and explosion-proof shields [15].

**Figure 1:**
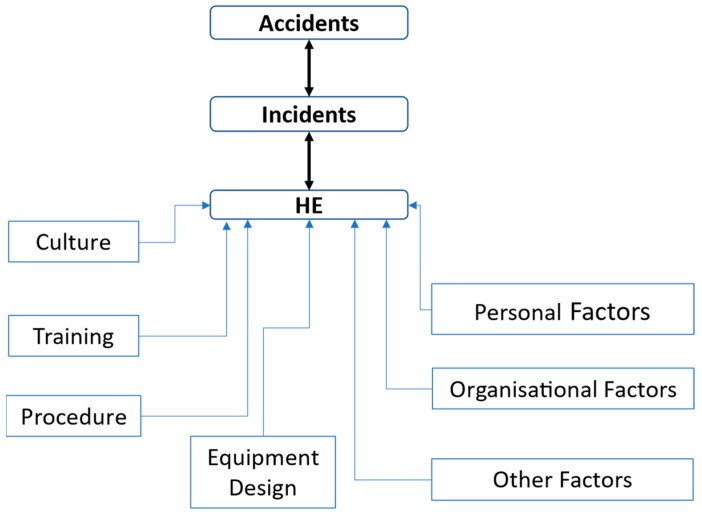
Contributing factors of HE.

There are many chances for experimentation with data science and AI approaches, such as ML, for industrial data analysis. ML may uncover insights concealed in event report patterns invisible to HFs or standard categorization methods. Automated classification may eliminate the need for people to interpret unstructured narrative material, and this could speed up event classification and, thus, solution development and deployment. Data science differs significantly in that it emphasizes preliminary analysis, forecasting, and visualization. AI uses a predictive model to foresee potential outcomes. “Data Science” describes multiple disciplines, including statistics, computer science, and industrial design. AI-driven systems can analyze data from sources and recommend what will and will not be provided. AI can also conduct in-depth analyses of users data and make predictions about consumer preferences, product development, and marketing channels. This paper proposes a prediction model for fireworks accidents and events of the FCEM in Sivakasi, Tamil Nadu. This also addresses the following objectives:

- Prediction of HE using ML models.
- Performance analysis of the data and recommended ML models to identify the optimal solutions. Suggestions for an ES based on the modeling performance for reducing HE-related industrial accidents. Figure 1 represents the influencing factors (IF) of HE.

Toxic release, fire, and explosion are the primary dangers associated with the FCEM. When it comes to potential harm to people and property, fire is by far the most significant threat. Among the most common forms of danger in fireworks is static electricity. These hazards may emerge when the solid’s surface and the chemicals come into contact. The health risk is merged into the root cause of the risk assessment model in the Risk Assessment of Safety and Health (RASH). As a general risk assessment model, this method was proposed for the construction industry but can be used in the FI as well. RASH could be a suitable method for predicting the risk in the FI as it involves handling and prolonged exposure to chemicals. In addition, RASH incorporates the likelihood of safety and health and vice versa for severity, whereas, previously, only the combination of rigor and probability was known as risk [16].

This paper is structured as follows: Section 1 describes the fundamentals of firework SMSs and measures and presents the problem statement of this research work. Section 2 provides the background of existing works. Section 3 is the proposed method of the fireworks industry disaster mitigation system. Section 4 explains the results and discussion. Finally, Section 5 concludes this research work.

## 2. Related Works

Regarding accident prevalence and magnitude, the FI is among the most vulnerable. Risk assessment, hazard identification, and the development of a fool-proof process requires detailed awareness of chemical compounds’ stability and explosive attributes, as stated by [17]. As a result, most fireworks SMS research is focused on the sensitivity and hyperactivity of individual components and materials. An early attempt to investigate what led to an explosion in the Italian FI was proposed by [18], who found that the manufacturing process lacked stringent controls and inspections mandated by a SMS for high-risk facilities. The lack of standardized equipment, tools, and manufacturing procedures, as well as an incomplete understanding of the thermochemistry and explosive nature of fireworks, are the leading causes of accidents [19]. To better promote a safe work environment, the author of [20] investigated the factors that caused casualties in the FI, finding that careless behavior, attitude, and safety hazards were significant contributing factors. Improved safety and reduced logistical support, storage, and manufacturing complexity result from a novel method for formulating highly explosive materials [21].

### 2.1. Human Error

Human factors (HFs) are usually crucial in workplace accidents. HE is “the failure to complete a given job or the conduct of a banned behavior”. A “near-miss incident” is one in which there is a possibility of a severe injury or property damage as well as a less severe injury or damage [22]. It is vital to remember that humans will make mistakes no matter how much training, experience, or talent; they must work efficiently [23]. Human reliability is influenced by the working environment, which is controlled by the design of equipment and management methods, among other factors. A thorough awareness of the types of errors is required. Errors occur when tasks are not adequately planned. Falls happen when jobs are not properly executed, such as when someone loses attention [24]. “This is a mistake if the purpose is wrong. This is a slip if the activity is not what was planned” [25]. Other types of HE include violations (a decision not to do something) and mismatches (someone is unable to perform something) [26].

Thousands of people work in the FI in Tamil Nadu, and many are exposed to hazards because of unsafe conditions. Accidents happened as a result. For example, during the recent explosion [27] at a fireworks facility in Virudhunagar, 20 employees died and 28 were wounded. This happens because the industry and people are not adhering to safety regulations when handling explosive chemicals. According to accident statistics, as much as 90% of industrial accidents may be traced to HE, suggesting a failure of the injured individual or a co-worker in following the SMS. Most of the previous study was based on unsafe acts and circumstances, with HE usually identified as the cause of accidents. However, no evidence exists that the fireworks mishaps were solely caused by HE.

The Factories Act of 1948 established safety standards for FI facilities. Housekeeping, protective clothing, employing women, and precautions were all addressed in this law. Since its founding in 1898, the Petroleum and Explosives Safety Organization (PESO), formerly the Department of Explosives, has been the nation’s nodal agency for regulating hazardous substances such as explosives, compressed gases, and petroleum [28].

### 2.2. Algorithms of Machine Learning

ML is becoming increasingly used as a tool for classification and decision-making systems (DMSs). For example, clinical-diagnostic decision support systems (CDSSs) have become increasingly popular in the medical profession in recent decades. These technologies improve clinical diagnostic testing accuracy and speed compared to traditional methods, which depend on the clinician’s observation, patient records, and data interpretation [29]. 

Today, ML is used for Medical Image Processing (MIP), Natural Language Processing (NLP) of medical documents, and Genetic Information (GI). Many of these areas are primarily about figuring out what is wrong, finding it, and making predictions about it. Currently, the extensive infrastructure of health care devices is in charge of classifying data. However, the infrastructure that supports these devices is often lacking, which makes it difficult to use these data effectively. Because health care information can be found in different formats, the data formatting method can be challenged and may increase background noise. By integrating specific clinical knowledge, patient information, and other health information, a CDSS aims to elevate health care provision [30].

■ By matching patient characteristics with a computerized clinical knowledge base and presenting the clinician with patient-specific assessments or recommendations, a traditional CDSS is meant to serve as direct aid to a clinical DMS.
■ Today’s most common application for CDSSs is at the point of care, where the clinician can incorporate the CDSS’s information or recommendations into their existing body of knowledge. Data and observations that would be difficult for humans to obtain or interpret are increasingly being used in the development of CDSSs.

### 2.3. Benefits of CDSSs

The ethical approval authority and code are listed for human/animal intervention studies [31].

- *Reduces Patient Safety:* The prevalence of treatment errors and adverse events.
- *Clinical Management:* Compliance with clinical procedures, reminders for follow-up, and treatment.
- *Low-Cost Measures:* Duplicate tests and orders, low-cost treatments, and tedious tasks are automated to reduce provider workload.
- *Administrative Performance:* Automatic selection of diagnostic codes, documentation, and note filling.
- *Support for Diagnostics:* Using patient data to make diagnostic suggestions and automating the output of test results.
- *Medical DMSs:* A DMS is delivered directly to patients via Electronic Medical Records (EMR) and other systems.
- *Workflow Optimization:* CDSSs can enhance and expedite an HER clinical workflow with improved data retrieval and presentation.

Similar inefficiencies are found in classification works conducted in other safety-critical industries (e.g., chemical, mining, aviation, rail, and automotive). ML methods are applied with little concern for the type of data, its structure, its quality, or the anticipated outcome. These approaches may function with new data [32]. In order to overcome the constraints of classic statistical models, ML approaches are now widely used in studies because of their superior predictability, time commitment, and educational value. In the past decade, ML has been used in the construction sector, occupational accidents, agriculture, academic classification, sentiment classification, and banking and insurance [33]. Applying HF ethics can aid in developing an automatic classification system that allows human input (unstructured data). The “system” must also be scrutinized regarding data quality, process and design patterns, human–machine interactions, algorithm output, and overall performance [34]. In some instances, ML may be the most effective classification method.

The coefficients of “a line-by-line combination of known template signature patterns” are designed to work for radio-isotopic classification using the original Fireworks Algorithm (FA). For software cost estimation, the FA will improve the feature subset. The objectives are to maximize the subset of features for high-voltage transmission line icing forecasting. Regarding feature and weight optimization, the weighted pattern recognition technique for imbalanced classification uses the FA. This FA applied the SVM’s parameters after benchmark datasets showed their performance. The twin support vector regression prediction model’s coefficient values will improve with the FA for basic oxygen steelmaking end-point prediction. The mission objective is to optimize the parameters of the multi-class SVM for recognizing abnormal control chart patterns. The least-SVM is evaluated with PSO for forecasting the short-term power load. The firework model trains the Feed-Forward Neural Network (FFNN) in its early phases to predict the agile software effort. For classification methods in processing health care records, many researchers used the FA to optimize the FFNN. For stock-exchange prediction, the FA is used in the initial phase of training the FFNN (which is then continued with the back-propagation algorithm); for classification, techniques in ML, PSO, and the supervised model are used to train FFNNs for detecting fireworks [35].

Regarding actual human input analysis, the type of data, the manner of entry, and the accuracy of the alignment are all important considerations. This method is used to improve the design of data entry systems. In some cases, such as when data collection is limited, automatic ML classification is more effective than manual classification [36].

According to the ML application literature, researchers should investigate various ML classification models before settling on one to apply [37]. In the short term, front-end analysis can predict ML’s success, how it should be used (alone or with other methods), and which classifiers to use [38]. This study investigates the dissection of safety incident reports in order to evaluate categorization algorithms that use intelligently produced input features as input features [39].

## 3. Proposed Methodology

### 3.1. Preliminary Factors of the SMS

Due to the low skill level among the workforce, better training in handling explosive chemical compounds is crucial for accident prevention. Storing the unfinished chemical mix in manufacturing plants is unacceptable, so chemical filling, rolling, and fuse fixing are completed on the same day [40]. Rubber mats should also be put down in manufacturing factories to reduce friction. Fires can start if raw materials or final products (gunpowder beads) meet pebbles or metals while drying. In addition, make sure to follow the safety measures that are classified below.

■ Maintain a well-trained and knowledgeable staff.
■ Preserve all valid licenses, authorizations, and safety checks.
■ Keep communication and safety on the display site up to date.
■ Use protective equipment and appropriate clothing.
■ Avoid creating potential ignition hazards by accident.

### 3.2. The Cross-Industry Standardized Procedure for Data Mining Framework

If this work needs a logical method for planning this research work, look no further than the Cross-Industry Standard Process for Data Mining (CRISP-DM) framework [41,42,43,44,45]. The methodology is reliable and well tested. However, we are evangelists for its functionality, versatility, and utility in resolving challenging business problems with business intelligence. Almost every client interaction has this as its golden thread. The CRISP-DM framework is an idealized timeline. In practice, multiple tasks are performed differently, and repeating actions is often necessary. The model does not consider all the ways data mining is performed. The accident data from 2000–2021 were used to predict HFs. CRISP-DM was employed to obtain the desired result. The objectives are developing a classification model for HF features and forecasting the outcomes. This research was organized into five portions using the CRISP-DM frameworks [46,47,48,49,50] described. Process models are independent of the industry in which they are used, and the methodology implemented [51,52,53,54,55]. Steps were taken in this research by CRISP-DM representatives. 

Business Understanding, the first stage of CRISP-DM, is about elucidating the business problem and giving the work a clear focus. In their haste to find data, analyze it, and try new techniques, analytical teams routinely fail to highlight the value of this precision. The CRISP-DM research work addressed some of these issues by defining a process model that provides an industry- and technology-independent framework for research works. For example, extensive data mining work is made more efficient, less expensive, more predictive, and easier to manage and speed up with the help of the CRISP-DM model. With 43% of the vote in the most recent KDnuggets Poll, CRISP-DM is still the most helpful approach for analytics, data mining, and data science research. On the other hand, CRISP-DM has not been modified for a very long time, so it is high time for a new approach [56,57,58,59].

### 3.3. Identifying Features of CRISP-DM

The reliability of CRISP-DM in a highly dynamic field is due to several factors:

(a) Data operators are strongly encouraged to keep the company’s vision in mind, which helps ensure that the research study will obtain valuable data. A standard error for experts is making their analysis an end rather than a means to an end, which is pointless for business. The CRISP-DM method guarantees that the company’s objectives remain central to the research work throughout its timeframe.
(b) A key feature of CRISP-DM is its iterative nature, which allows for continuous monitoring of the research project’s development concerning its policy objectives. One benefit is a reduced chance of discovering that the business objectives were not adequately met at the conclusion of the research. It also implies that the system’s decision makers can modify and adapt the goals considering new information.
(c) Technology and problem neutrality are hallmarks of the CRISP-DM approach. As for the analysis tool and the data mining issue, people can use whatever they want. Research works of all shapes and sizes can benefit from the study results by CRISP-DM.

#### 3.3.1. Step-by-Step Information about the CRISP-DM

(A) Business Understanding.
(B) Data Understanding.
(C) Data Preparation.
(D) Modeling.
(E) Evaluation.
(F) Deployment.

##### A. Business Intelligence (BI)

The BI stage aims to learn about the issues the company is trying to address. The FCEM identifies the following as critical activities during this stage:

*Identify the Business Goal and Challenge:* To define a business’s success criteria, one must first understand what problems are solved from a business perspective and what customers want (Key Performance Indicator (KPI)). 

*The Current State of Things:* It is necessary to evaluate the research’s resources, needs, risks, and cost–benefit ratio. 

*Set the Research Interests:* The criteria for success from a technical data mining standpoint. Use it to explain the proposed basis for the threshold: model benchmarks or availability time.

*Preliminary Research Design:* Attempt to create a detailed plan for each research phase and the tools used.

However, much research has been performed to improve industrial SMSs. Most studies have employed traditional statistical methodologies, but with ML and new superior technologies, these predictions are identified more precisely, assisting the aviation industry in determining the correct cause. When creating projections, these additional features must be considered [60].

Previous research had faults because of the inaccuracy of the used models and attributes. A classification system is both highly accurate and efficiently implemented. The two leading solutions are as follows:

■ The first step is to classify these HF-related properties.
■ Better analysis with ML models and historical data.

##### B. Data Preparation

Needed to understand the data to solve the business problem identified in the BI phase. Following the FCEM’s recommendations, this stage might include the following:

*Collect Data:* The required data are unavailable outside the organization; collect it or use free data to construct our research.

*Describe Data:* Check the data format, rows and columns, field names, and features.

*Explore Data:* Visualize data relationships and be creative.

*Verify Data Quality:* What is the quality of the data? How many values are missing? Have the data been collected properly? Check the data quality.

The historical dataset of past incidents and accidents for 21 years was collected from web sources, police stations, fire services, news articles, and questionnaire surveys gathered from the workers. The data to be analyzed include psychological factors, HFs, and organizational factors.

Age, education, experience, working time, fatigue, stress, training, supervision, infrastructural facilities, job factors, knowledge and understanding, personal safety, and highly hazardous situations are just some of the psychological factors that can affect workers. Additionally, the mode was used to compute the average and the method of the attributes, and only the age factors were deleted because they were not substituted in any other method.

**Figure 2:**
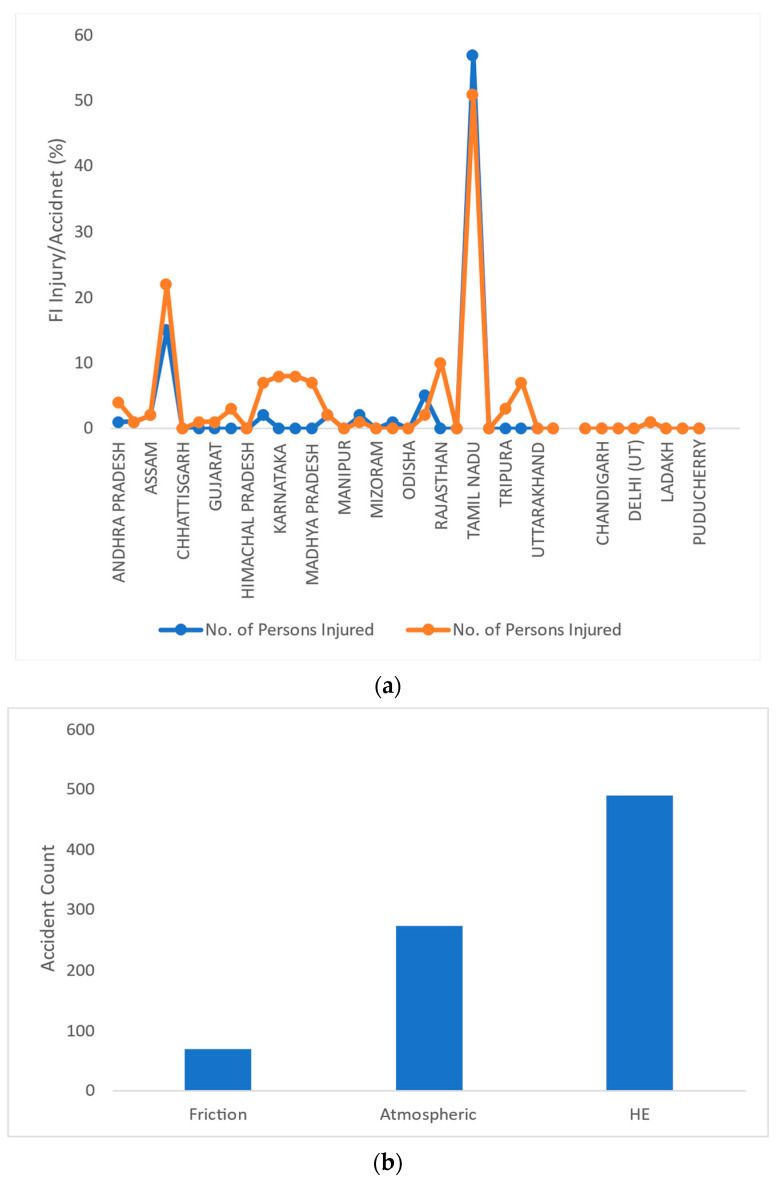
(**a**) Accidents range from 2021; (**b**) number of fatalities due to accidents [https://www.indiastat.com/data/crime-and-law/total-fire-accidents/data-year/2021] 31 December 2021.

The data included in this report were collected from state and local statistics. Figure 2a shows an upward trend in accidents in 2021. In addition to the loss of human death, infrastructure, repair, and rehabilitation of damaged structures result in a loss of productivity. Additionally, these incidents harm surrounding manufacturing firms and the public. Figure 2b represents the IF for past accidents and incidents.

Modeling is used to divide the IF of FI accidents into three classes based on the tasks: (i) an HF event—the result of HE in all processes; (ii) an industrial infrastructure factor; and (iii) environment states (humidity, temperature, and pressure)—one that is not associated with or caused by HE. 

Currently, classifying HF incidents necessitates continuous monitoring of manufacturing processes. Therefore, while making predictions, the data are divided into two sections so that the training model is functional for 80% of the data, and the testing model is used for the remaining 20%.

After that, the Comma-Separated Values (CSV) file needed for our research is extracted and imported into Python for further analysis. Pre-processing the data is the next step before classification. Then, the categorization results are compared to see which is the best, and the results are graphed. 

##### C. Data Pre-Processing

Pre-processing raw data is required for use in an ML model. Making an ML model requires this first and most crucial step. A total of 1300 records from the FI’s accident and incident database were used for this study, including 150 unique features. Specifically, for this study, we looked for 110 HF qualities. This function was used to remove different anomalies, such as characters in statical attributes, and vice versa from the dataset. The fact that these data were retrieved from different file formats means these abnormalities were present across all features. This was accomplished through the usage of Python. Managing missing values in data is a significant component of pre-processing. It was determined that several missing values in the library were “missing.”

Pre-processed data are used for feature extraction. Comparing three feature selection methods is helpful in this study, with the most suitable method chosen for model implementation. This research includes many classification methods, such as rule-based and ML algorithms. Algorithms such as NB, RF, XGBoost, and SVM predict how HFs would influence the FI.

- Data Selection: A DMS was run on the dataset, columns, and rows to implement. The business dilemma should affect how to filter data.
- Data Cleaning: This is a data-checking task. Cleaning data requires preparation, understanding, and a reason to correct or impute data.
- Feature Engineering: What may or may not interest feature engineering? Use your imagination when coming up with new information based on old data.
- Data Integration: Combining existing datasets to create a new one. Obtaining the dataset from information sources may be more challenging, but it is possible.
- Data Formatting: When necessary, data are formatted. Change a classification value to a numeric one, for instance.

##### D. Modelling

The modeling phase is where a lot of the action happens, so it attracts many data subscribers. In contrast to the previous phase, however, this one will not last as long. The proposed ML model to address the study questions will be developed during this stage. 

*Possible components of the task as described by the FCEM are:*

*Model Selection:* How to decide which ML algorithms to test. This work needs to try out several models to find the best fit. Therefore, knowing the rationale behind this algorithm choice would be helpful.

*Test Design:* Organize the data into training, test, validation, and cross-validation sets before designing the modeling test.

*Model Development:* Run the model with the collected data. It may take a long time, depending on the data size and experimental design. To ensure a successful outcome, questions such as “Is the model develop possible in the business?” and “Are the resources needed to develop this model expensive?” should be weighed alongside technical requirements.

*Test Model:* Choose the best business models and set technical success metrics.

##### E. Evaluation

Model evaluation differs from evaluation. The business indicator model and the next steps are estimated during this phase. The phase responsibility of the FCEM comprises the following:

*Outcomes:* Would the business success criteria be met using this model? At this point, it should detail how this model will benefit the company.

*Review:* Is process check not enough? Time? Are all steps taken? Summarize and correct findings. The preliminary iterative process of the data science study does not have to be completely perfect. The company wants to learn from HE.

*Decide What to Do Next:* Determine whether the model is ready for deployment, requires additional iterations, or creates new work based on the previous tasks.

##### F. Deployment

If the user cannot obtain the model result, it does nothing good. This phase may seem too much for a fresher, but a realistic idea will impress the company. This task for the FCEM phase is:

*Deployment:* In what way do you envision the model being used? In what form is the outcome communicated to the beneficiary?

*Opinion and Maintenance:* How will the monitoring and maintenance plan be structured? As with any other phase, output monitoring and maintaining the model’s quality are crucial.

*Findings:* Create the summary report, develop the demonstration, and attempt to present it to someone to conclude the work.

*Analysis:* Analyze the research by considering what performed well, what could be improved, and what there was trouble finding.

### 3.4. Objective of Risk Evaluation for Business Impact Analysis (BIA)

One way to use the expertise of people who know a lot about a particular topic is through using ES. It is based on the idea that information is available. In order to aid business DMSs, BIA performs tasks such as data collection, database management, data processing, data aggregation, and data application. BI is improved by information management. Evaluations of risk highlight potential issues and their outcomes. BIAs evaluate the failures in essential operations. Risk is assessed methods. Qualitative risk analysis is a more direct and practical method. Based on perceptions of the seriousness and propensity of its effects, qualitative risk analysis rates or scores risk. On the other hand, quantitative risk analysis calculates risk based on already-known information.

Human-social, physical, economic, and environmental vulnerabilities and their direct and indirect losses are listed below in Table 1.

#### 3.4.1. (a) Rule-Based Approach

Any ML technique that discovers, obtains, or evolves “rules” to store, manipulate, or apply is under the umbrella term of “rule-based machine learning” (RBML) in the field of computer science. Rule-based classifiers can also make classification decisions using a series of “*If. Else*” rules. These classifiers create descriptive models because their governments are simple to understand. The methods for ML were chosen based on the findings in the literature [60]. The most common algorithms for supervised text categorization in RB systems are NB, SVM, and RF. NB and XGBoost are recommended for mixed data with continuous inputs [62].

- Rule-Based System Architecture

**Figure 3:**
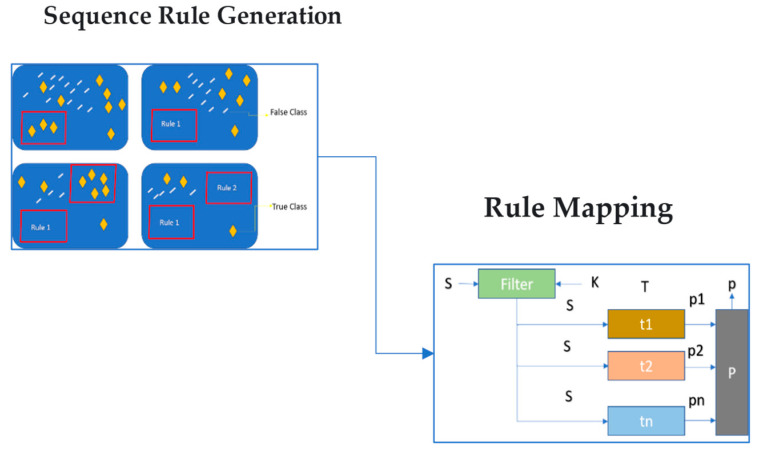
Rule-based model. Yellow represents True Class, Red indicates Rule applied.

The structure of the system is divided into two distinct tiers. A pattern matcher is used in the second level, but only after being filtered out in the first. The system’s output is the predicted score of the worker’s appropriate response. Figure 3 depicts a step-by-step representation of the system.

Step 1. There are two inputs to the filter stage.
Step 2. The first input is the worker’s response S = {S_1_, S_2_...S_k_}, and the second is a set of keywords, ‘K.’
Step 3. The result is a set of sentences S′ = {S_1_, S_2_,... S_n_} for every possible path T = {T_1_, T_2_,... T_n_}.
Step 4. Depending on the original worker’s sentence length, each list S_i_ may contain ‘0′ or every word in it.
Step 5. If a keyword appears in a sentence in Si, that sentence is added to the Si sentence list.
Step 6. The input of the pattern box is a string of sentences, and each pattern may be unique.
Step 7. If the sentence passes the test, the output is true; otherwise, it is false.
Step 8. The result, ‘p’, is the cumulative result of all the experimentations.
Step 9. This system can grant a three-point maximum of points.
Step 10. The system’s final output is ‘p’, which ensures the final score is [0, 3].

Assume that the classification problem contains ‘*c*’ classes in the ‘*n*’ dimensional pattern space, and there are ‘*p*’ vectors *Xi* = [*xi*_1_, *xi*_2_, …, *xi_n_*] (*i* = 1, 2, …, *p*). An *If-Then* rule is presented as finding the recommendation.

When choosing a property that is not valid for classification, misclassification may result. Misclassification happens when all classes, groups, or categories of a variable have the same error rate or probability of being false. To reduce misclassification, distribute samples evenly within each category [63]. Take a small value for the initial learning rate when defining the parameters to improve accuracy. Training, validation, and test data accuracy comparisons come first. Assigning each ‘X’ to the group with the smaller integrand value in the preceding exponential function effectively reduces the success rate of making a mistake, or ‘p’ (error). The value of ‘x’ should be placed in class C1 if the probability of placement in C1 is greater than the probability of sequence in C2 for that value of ‘x’. This work chooses the misclassification error (ME) as the fitness evaluation, which is defined below, Equation (1).

(1) $$\mathrm{ME}=\frac{Number\;of\;Misclassified\;Industries}{Number\;of\;Industries}$$

**Figure 4:**
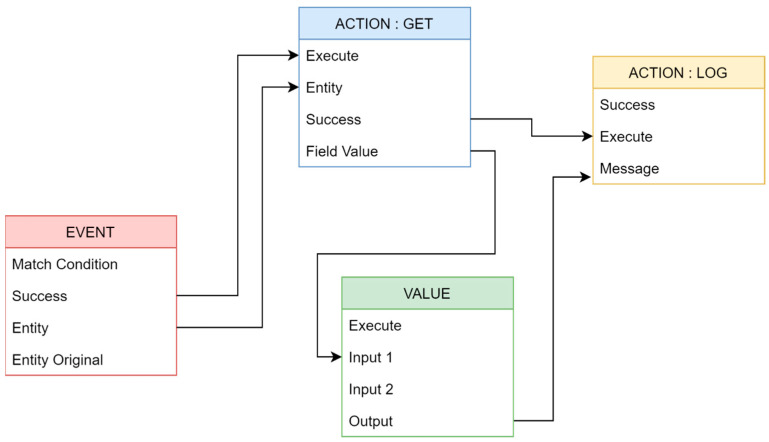
Configuration of the If-Then-Else flow of misclassification.

The selection of inappropriate features for classification is a potential source of misclassification. It is supposed that there is misclassification when there is an equal chance of error across all classes or clusters of variables [64]. From this confusion matrix (CM), this research derives classification accuracy by dividing equal cells by equal correct cells (TP + TN). The nodes (boxes) of an *“If-Then-Else”* rule have outputs connected to the inputs of other nodes, creating a graphical layout. The box in Figure 4 shows the configuration options for each node. Nodes are classified into Event, Action (GET and LOG), and Value.

The objective is to identify the optimal process parameters in Figure 4 that reduce the ME to the maximum extent [65]. The classification accuracy (CA) ratio is the number of classified companies divided by the number of companies. ME + CA = 1 Equation (2).

(2) $$\mathrm{CA}=\frac{NumberofClassifiedIndustries}{NumberofIndustries}$$

From 2000 to 2021, 834 “important” operational values (HF = 490, non-HF = 344) were active in algorithm testing. Continuous age, experience, training, personal factors, categorized drop-down selections, and open-text narratives are all included in event reports [66].

Step 1. R → set of rules generated using a training set;
Step 2. *t*-test record;
Step 3. W → class name to weight mapping, predefined, given as input;
Step 4. F → class name to vote to map, generated for each test record, to be calculated for each rule ‘r’ in T check if ‘r’ covers T;
Step 5. If so, then add W, F of Predicted Class;
Step 6. End for;
Step 7. Output the class with the highest rated vote in F.

#### 3.4.2. (b) Random Forest (RF)

This algorithm combines multiple DT performances to produce more reliable and precise models. In order to increase the accuracy of a community-based DT, potential overfitting is avoided by reducing the variance in the study system [67].

RF creates Decision Trees (DT) for variables. It is a dark box since it is difficult to tell how it works inside the model. Studying deep DT and processes would be difficult. Individuals could learn from bagged data with random features [68]. So, the RF can help us compute feature relevance. Let us first look at how an RF works. A sub-feature protects the decision paths from the node to the last leaf. Individuation and prejudice influence prediction (mean value of top-most region covered by training set). DT is used to classify small data samples and then average them to improve prediction accuracy while controlling overfitting. RF is a sort of estimator that uses this method. These data predictors would have no scaling limitations, outliers, or missing values. DT prediction function is defined as Equation (3):

(3) $$f(x)=Cfull+\sum_{m=1}^{M}Contrib(x,k)$$

M—tree leaf count, k—feature count, and Contrib (x, k)—*k*-feature contribution in feature vector ‘x’. Let us now examine the RF prediction, which is the average value of its tree prediction. So, RF prediction function Equation (4):

(4) $$f(x)=\frac{1}{J}\sum_{J=1}^{J}f_{j}(x)$$

RF provides the probability of belonging to a class for a classification problem. The possibility must convert the distance to the boundary using SVM if needed. SVM outperforms RF in most cases [69].

#### 3.4.3. (c) Gaussian Naïve Bayes (NB)

Data features with continuous values are assumed to be drawn randomly from a Gaussian distribution (normal distribution). Cost estimation data distributions with this technique are simple and transparent [70].

The Bayes theorem is the basis for the NB probabilistic ML algorithm for many classification functions. The Gaussian NB function extends NB. The NB model is measured to be a generative model. Each class is assumed to have a Gaussian distribution in NB. NB differs from Quadratic Discriminant Analysis (QDA) because it takes the independent features, resulting in a diagonal sample covariance matrix. NB is a binary (2-Class) and multi-class classification algorithm [71]. When describing the method, it is easiest to understand if it uses binary or categorical input values. The NB model is simple to construct for available datasets and highly effective. Along with simplicity, NB outperforms even highly sophisticated classification methods. Bayes’s theorem tells us how to figure out the posterior probability P(c|x) from the prior probabilities P(c), P(x), and P(x|c), Equation (5).

(5) $$\begin{array}{l} Posterior\_\mathrm{Pr}obability⟦P(c|x)⟧=\frac{Likelihood⟦P(x|c)⟧\times Class\_\mathrm{Pr}iority\_\mathrm{Pr}obabiloty⟦P(c)⟧}{\mathrm{Pr}edictor\_\mathrm{Pr}ior\_\mathrm{Pr}obability⟦P(x)⟧}\\ P(c|x)=P(x|c)\times P(x|c)\times ...\times P(x|c)\times P(c) \end{array}$$

Above, the prediction of class (c, target) given the coefficient of determination ‘x’ is P(c|x) (x, Attributes)

The occurrence, denoted by P(x|c), is the probability that the predictor is correct given the class ‘c.’

P(c) is the prior probability for the class.

P(x) is the previous probability of the predictor.

#### 3.4.4. (d) Support Vector Machine (SVM)

SVM is a novel technique for classifying linear and non-linear data. This algorithm is used because of its high generalization performance. It is effective on high-volume datasets, reduces the number of processes involved in learning, and makes memorization less likely, but the learning time is improved. An SVM is an algorithm that operates in the following manner. It renovates the unique training data into a higher dimension via non-linear mapping [72]. Surrounded by this new dimension, it searches for the linearly optimal separation hyperplane (i.e.,) a “decision boundary” for classifying tuples. A hyperplane can always divide data from two classes when an appropriate non-linear mapping to a necessary high dimension is used.

The SVM is the most often used classification model in supervised ML for classification tasks. Because this model is based on a kernel-based method, it is used with a subset of training data. The non-linearity in a dataset is distributed using a kernel in ML. Adding a user-specified kernel function (similarity) gives the dataset an extra dimension, allowing it to be subdivided along a linear higher dimensional space. Kernels inherently transform conceptually distinct data into removed data, allowing a linear classifier to be applied to a non-linear problem [73].

In order to apply a linear classifier to a non-linear problem, kernels transform data that are inherently inseparable into data that can be easily separated. The original vectors are predictable in a higher dimensional space, where they can be linearly separated, which is helpful when dealing with non-linear problems. Kernel methods do not use explicit transformations (x) to represent data. Instead, they only use pairwise similarity comparisons between the original data observations (with the actual coordinates in the lower-dimensional space). These transformed kernel methods represent data only through pairwise similarity comparisons between original data observations (x) instead of explicitly applying transformations (x) (with the actual coordinates in the lower dimensional space).

Its central purpose is to increase the margins of these hyperplanes employed in the different classes of samples [74]. The SVM determines the hyperplane using support vectors and margins. The classification error decreases as border separation and distance between parallel hyperplanes increase.

#### 3.4.5. (e) XGBoost

This is a DT-based community learning algorithm similar to traditional gradient enhancement models. This is innovative because it was determined in 2016. Scalability is the main differentiating factor, allowing for rapid learning via parallel and distributed computing and efficient memory usage. Overfitting and bias have been eliminated [75].

**Figure 5:**
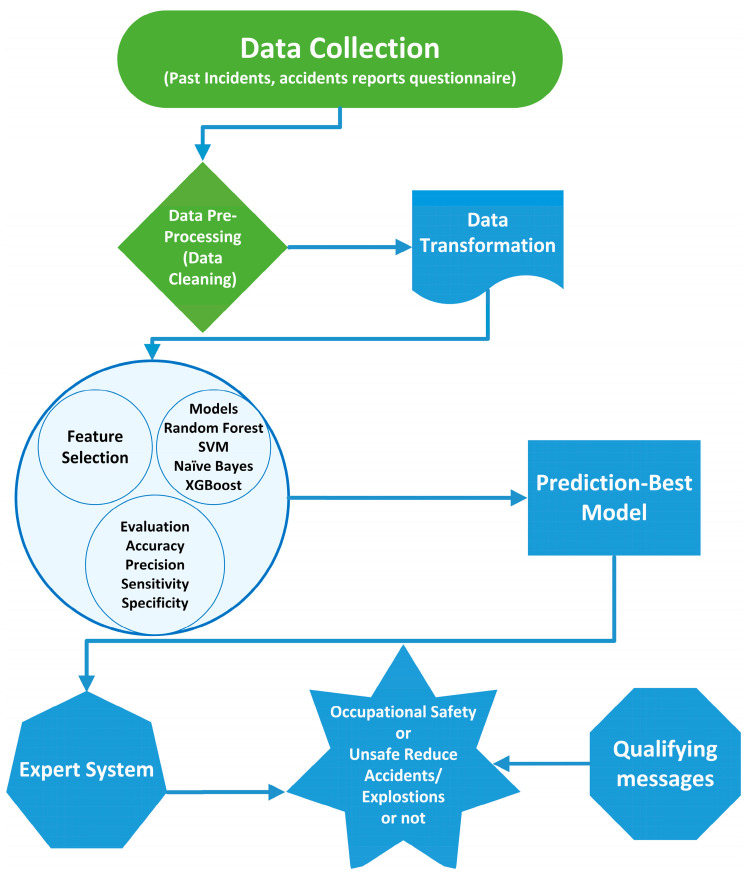
Flow diagram of the prediction.

XGBoost is a commonly used supervised learning algorithm. It uses a highly scalable training strategy that minimizes overfitting and successively builds small DTs to produce reliable results. The most well-known and hyped-up model is XGBoost, which integrates tree-type learning with linear models and is used in parallel computing. It is also the most widely used model. The Gradient Boosting (GBM) technique was created for strong predictive power. Because the approach requires only one DT per model, usage was restricted. However, even small models take a long time to train. So, a new method called XGBoost came along and revolutionized GBM. Data are structured in XGBoost to optimize lookup times, and several cores generate separate trees, improving model performance by reducing training time (Figure 5).

- Prediction from XGBoost

To predict a new data point, this work will use structured trees or models to obtain all values to solve Equation (6)

F_2_(x) = σ (0 + 1 × h_1_(x) + 1 × h_2_(x))) (6)

The resulting value of F_2_(x) is the prediction from the XGBoost model.

In comparison to traditional GBM, XGBoost has more rigorous regularization. XGBoost uses regularization (L_1_/L_2_) to enhance model generalizability. In comparison to GBM, XGBoost provides superior performance. It has a fast-learning curve and is trained parallel across clusters. Numerous benchmark studies and Kaggle competition victories attest to XGB’s superiority in multiple cases [75,76,77,78]. GBM is very common, and XGBoost is parallelized and runs faster than other methods, making it more successful.

### 3.5. Evaluation Metrics

Multiple methods of evaluation and metrics are employed in this study to assess the proposed method’s effectiveness in predicting the HFs influencing the fireworks accident training set. Accuracy, specificity, sensitivity, precision, and F1-score are metrics used in Equation (7).

(7) $$\mathrm{Accuracy}=\frac{TP+TN}{TP+FP+FN+TN}$$

- True Positive (*TP*)—Indicates that the prediction is positive and is typically true.
- True Negative (*TN*)—Indicates that the prediction is negative and is typically true.
- False Positive (*FP*)—Indicates that the prediction is optimistic and is typically untrue.
- False Negative (*FN*)—Indicates that the prediction is negative and is typically untrue.

### 3.6. Fold Cross-Validation

Cross-validation (CV), called k-Fold, is similar to the well-train–test split. Cross-validation is referred to as k-Fold cross-validation. When using k-Fold cross-validation, multiple (k) train–test sets are used instead of one. A k-Fold CV entails performing the training and testing steps a total of ‘k’ times. The reasoning is that if only one train–test splits, this model may perform exceptionally well on a subset of test data that are easy to predict but improperly on actual test data. For example, suppose this research divides the training data into four equal parts in a 5-fold CV. First, this work selects a subset of the five to serve as the test set, while the other four are used for training. The remaining portion is the validation set, while the remaining four become the training set. For the subsequent five iterations, this work will use a different component from the original location of features to see how well this model holds up. To a significant extent, k-Fold CV can also help us overcome overfitting.

### 3.7. Effect of Physical, Psychological, and Organizational Factors (OF)

The framework for the research is called the “research design,” and it tells us how to collect, measure, and analyze the data. So, this model is a plan for what the researcher will do, from writing hypotheses to looking at the results. The research design is the research plan, structure, and strategy to find new ways to solve problems and reduce differences. To achieve its aims, this quantitative researcher employed a questionnaire method.

A. Collection of Data: This research was planned to determine how much employees in the fireworks and match industries in Tamil Nadu interact with workplace safety and health data. The term “survey” describes an approach to research in which data can be collected through survey responses, interviews, and direct observation of the topic of study. This study’s primary data collection tool is a questionnaire with an interviewing methodology. A questionnaire is an organized list of independent investigations about the research issue to which the study participants are asked to respond. The collected data are tabulated and statistically analyzed to present the findings and presumptions in the final stage. It is performed with the aid of statistical analysis software. The subject of this study is the information literacy of employees in the fireworks and match industries in Tamil Nadu and their effects on workplace conditions and safety measures. Data collection is performed with an array of tools. The significance of each tool varies depending on the situation. The following methods and instruments were used in the present investigation:
B. Methods of Observation: Observation was used to see whether the survey participants’ information was objective. The investigative reporter went to the office over several days and during different business hours to see how the company works and what resources and services it offers, such as orientation or training programs for information literacy.
C. Area Under Study and Sampling: The respondents from the information literacy research study on working conditions and safety measures in the fireworks and match industries in Tamil Nadu were investigated. Based on the “Purposive Sampling Method,” test results from the population were selected. Workers in the fireworks and match industries in the Tamil Nadu districts of Virudhunagar and Thoothukudi comprise the study sample. According to reports, the districts of Virudhunagar and Thoothukudi produce approximately 70% of India’s FI and matches. FI and matchmaking thrive in the city’s hot and dry climate. Almost 90% of fireworks were produced in 2011 in the Virudhunagar and Thoothukudi Districts of Tamil Nadu, India, which also used 500 match factories and over 9500 FI facilities. Workers from the fireworks and match industries made up the defined population used for sampling. The number of people included in this sample is 443 out of 500 defined samples. In this study, first-hand information and data from different sources were used. Using an interview plan and a clearly defined questionnaire, primary data were collected from the people who responded. This work uses random sampling to collect data to ensure good results.
D. Match Work Industries: Three industry sectors make up the production of wooden matches in India: (i) the large-scale mechanized sector, (ii) the small-scale handmade sector, and (iii) the home-based business industry.

The handmade, small-scale (67%), and cottage (15%) sectors of the matches produced in India account for nearly 82% of the total production in the districts of Virudhunagar, Thoothukudi, and Tirunelveli. There may be more than 500 match work industries, according to estimates.

E. The Fireworks Industry: Approximately 90% of India’s fireworks are made in the Virudhunagar, Thoothukudi, and Tirunelveli districts. The business grows to be very financially beneficial for the employers.

### 3.8. Analysis and Interpretation

After data collection, the questionnaire responses were reviewed for accuracy and coded. The database was created by entering the coded data into a computer. Using the appropriate statistical methods, the tabulated data have been analyzed. To verify the predictions, suitable statistical methods have been used. Data manipulation has been performed using the Statistical Package for Social Scientists (SPSS) software (version 27) and MS-Excel Data Analysis.

### 3.9. Tools Used for Statistics

For this study, 500 participants from the fireworks and match industries in Tamil Nadu were chosen to fill out a questionnaire. The scheduling of interviews was performed using the interview method, and the finished schedules were collected for review. The techniques were used after the research questions and the research based on the data collection. The statistics, such as frequencies with good and cumulative percentages, were created first in the analysis section to conclude. In the current study, the following statistical tools were implemented:

- Percentage (%),
- Mean, and
- Standard deviation (SD).

Percentage analysis is a precise and effortless way to see how the study participants are spread among the different groups. The data were analyzed and presented in the following categories based on this study’s objectives: environmental, psychological, and organizational factors. 

### 3.10. Personal Factors and Workplace Environment Awareness-Percentage Analysis

The survey participants put much value on the light sources, air flow, firefighting equipment, emergency exits, and regular maintenance of electrical instruments that the industries give them. The participants also felt that the emergency light, fire suppression system, smoke detector, and flammable vapor storage were insufficient. Considering the overall feedback provided by respondents, it can be concluded from Table 2 that 37.02% of those surveyed believe the workplace environments are perfect.

### 3.11. Analysis by Percentage: Effects of Physical Factors

An overview of the effects on the physical environment. Table 3 data show that most workers, 4.987%, never reported experiencing a lump in their throat. Only a small percentage of workers, 4.784%, experience high blood pressure, but 76% have never experienced it. Despite stress at work, 4.671% of workers never get headaches, but 49% get headaches often. Infrequent spells of heat or cold were experienced by 55% of the workers. The data also indicate that employees occasionally face back pain and general weaknesses of the human body. Muscle pain was a symptom among workers at 3.795% frequency. The heart was thumping for 49% of people almost constantly. While working in the industry, an estimated 2.871% of workers observed tiredness almost constantly. Those with high blood pressure and have abnormalities in their throats account for 4.784%, the result of the workplace’s extensive use of chemicals and dust.

### 3.12. Impact of Physical Factors on the Test of the Theory

Men and women have no significant differences in how physical factors affect how well people can read and write in Table 4. 

### 3.13. Analysis by Percentage—Effects of Psychological Factors

The result of people working in unsafe workplaces has no psychological effects that they might think they would. However, there are times when they are forgetful, and 71.19% feel good. Sometimes they experience poor concentration during work, which is worth mentioning in a hazardous industry. It can be deduced from Table 5 that 24.54% of survey participants have workplace problems, while nearly 78.19% of survey participants claim to have never felt the impact of psychological workplace factors.

### 3.14. Verification of Hypothesis—Impact of Psychological Factors

There is no significant difference between the effect of a psychological factor on the gender of respondents and their level of literacy (Table 6).

### 3.15. Percentage Analysis—Impact of Organizational Factors

Most respondents never experienced feelings of being alone (99.19%), prejudice 91.19%, a lack of confidence at work 90.39%, a decrease in personal connections 88.38%, and or isolation from society 91.97%. Only a small percentage of those surveyed report feeling constantly confident (90.19%) or unconfident (65.33%). The respondent’s classes are shown in Table 7. Self-confidence significantly impacts survey participants more than mistrust. The respondents, in contrast, never experienced a sense of isolation, prejudice, or a lack of confidence at work. In total, 73.18% of the people surveyed said they had never observed OF at work.

### 3.16. Impact of OF: Testing the Hypothesis

There is no significant difference in the literacy level and impact of OF at the gender level of respondents (Table 8).

## 4. Results and Discussion

### 4.1. Rule-Based Approach Classification

Table 9 shows the accuracy values of the rule-based approach conducted using a collection of attributes in the dataset. 

An accuracy rate of 82% was achieved for HF and non-HF incidents, respectively. Rule-based detection events accounted for an average of 17% of all measures, a significant decrease from current practice (human review of all occurrences).

An adverse finding for the employer and the employees is that the worker quality in learning and understanding new information needed is below 50%. Therefore, businesses must develop a transparent environment to improve learning, processing, and DMSs. As they deal with highly flammable goods, awareness among all survey participants that their workplace environment is hazardous is 100%. Most respondents trust that the workplace environment is unsafe and may result in emergencies because they work with highly flammable raw materials.

As per the current FI working environment survey, 83.9% of participants said they experienced an accident, 26.1% felt a public health risk, 74.8% felt neck, leg, and finger movement problems, 27.5% felt eye problems, and 21.8% felt other issues. The respondents, 35% and 34%, respectively, equally preferred the three concrete variables of information that need to be predicted—physical, psychological, and OF. With a slight difference, social health is preferred by 31% of survey participants, guided by psychological and physical health.

### 4.2. Cross-Validation

When evaluating the models, the train and test sets approach and the k-Fold CV model were ideal as evaluation methods—CV based on stratified k-Folds. The data can be separated into train and test sets using the provided indices. This object for CV is a k-Fold modified version that returns divided into two class folds. The CV method for classification issues has been expanded upon by the stratified k-Fold CV. The class ratio remains constant throughout the k-Folds at the same level as in the original dataset. To assess the efficacy of ML models on a small dataset, researchers often employ CV, a resampling procedure. A parameter named ‘k’ specifies the number of groups a given training dataset is divided into. In this context, the term “k-Fold CV” represents the method. In order to create the folds, the proportion of values close to each class is held constant. Compared to traditional k-Fold CV, stratified k-Fold CV uses a probability sampling method instead of a random one.

#### K-Fold Cross-Validation Trains and Validates the Test Model

Step 1. The data are divided into “training” and “test” sets.
Step 2. Then, k-Folds are used to divide the training dataset.
Step 3. The (K-1) Fold is the training fold when K is large.
Step 4. Validity is measured by a 1-Fold increase.
Step 5. The model is tested using K-1 Folds of training data and one-fold of validation data. The model’s performance is logged.
Step 6. Each k-Fold is used for validation until the process described above (Steps 3–5) is frequent. Because of this, k-Fold CV is the name given to this method.
Step 7. Once all K models are recorded in Step 5, calculate the mean and standard deviation of model performances.
Step 8. For other hyperparameter values, Steps 3–7 are repeated.
Step 9. The hyperparameters that produce the best mean and standard model scores are chosen.
Step 10. Step 2 entails training the model with the training dataset and calculating the model’s performance using the test dataset (Step 1)
Step 11. Here, the diagram represents (Steps 2–7). This summarizes the idea behind k-Fold CV when K = 10.

**Figure 6:**
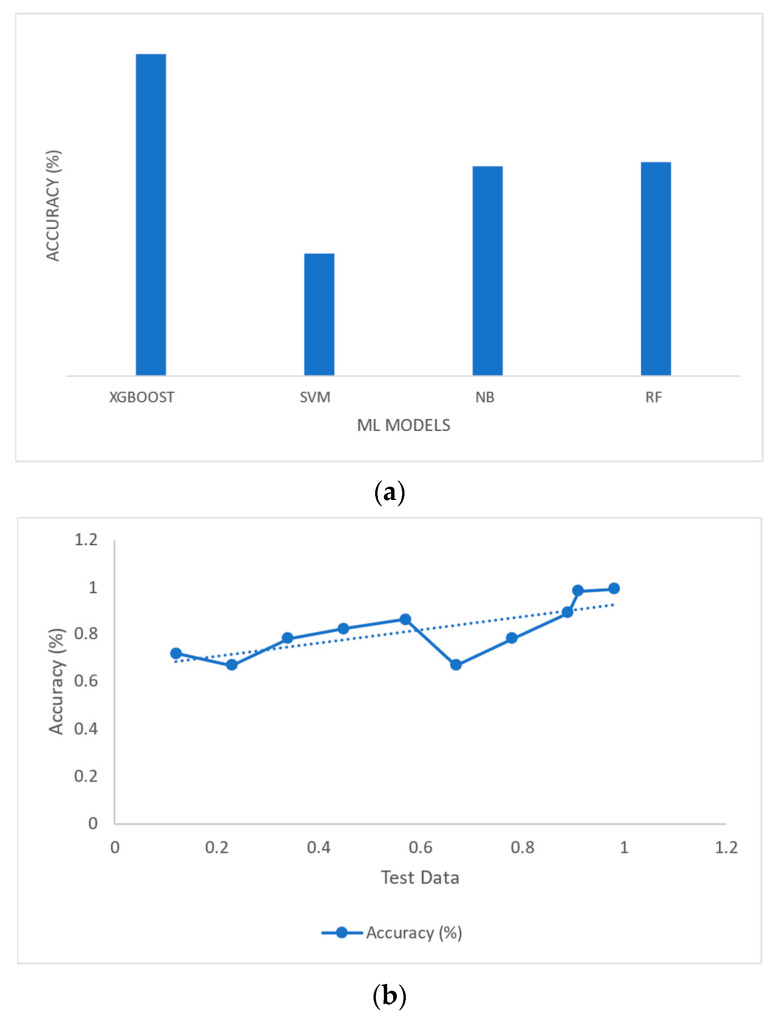
(**a**) Stratified k-CV; (**b**) k-Fold 10 CV.

Figure 6a,b compares two validation procedures: stratified k-Fold and 10-Fold CV. In stratified K-10, the output of the models XGBoost and RF was better compared with those models obtaining 92.75% and 91.53%, respectively, whereas the models SVM and GNB achieved 90.3% and 91.2%, respectively.

### 4.3. SVM

**Figure 7:**
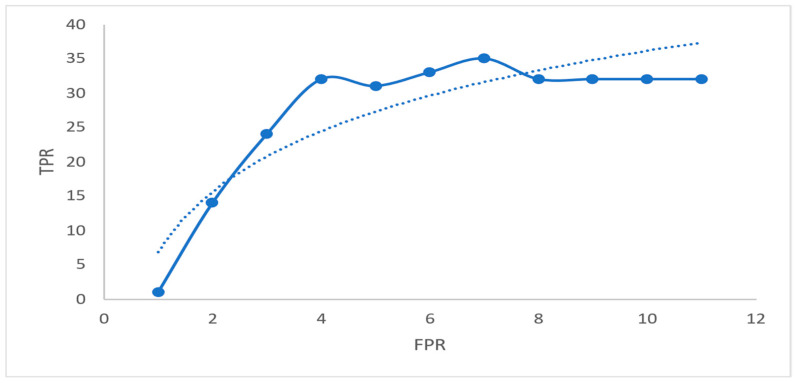
AUC result.

The k-Fold CV is to be tested from k1 to k9. Figure 7 illustrates the CV of SVM; the accuracy is not satisfactory. In k-Fold 10, CV gives a better accuracy of 92.85%, a sensitivity of 90.05%, and a specificity of 91.45%.

### 4.4. RF

RF used the parameters ‘ntree’ and ‘mtry’ for this algorithm’s execution. Three is the number of ‘ntrees’, and ‘mtry’ is the number of randomly chosen variables. This model’s most considerable ‘mtry’ value is set to 140. However, this had no impact on the categorization outcomes. An area Under the RoC Curve (AUC) of 0.890 was initiated at a confidence level of 92%. In order to obtain the best results, the RF model’s best training model was used to lay this AUC. Integrate q = f(p) between p = (m, n) to find the AUC between p = (m, n). It is possible to determine this region using integration within a specified range. According to Table 10, the performance metrics are registered.

### 4.5. Performace Evaluation

**Figure 8:**
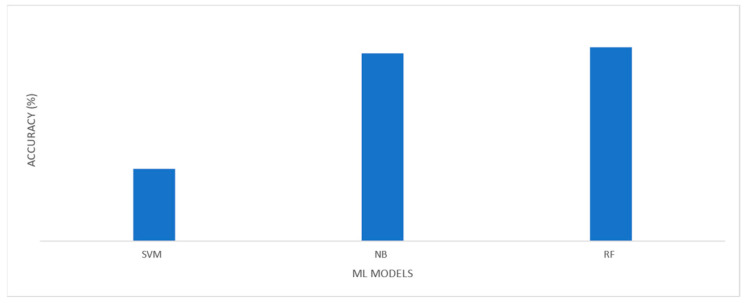
Performance measures of the proposed model.

Their accuracy measures the effectiveness of these four model classifiers. Table 11 provides an accurate description of each model. Among the currently available models, XGBoost’s CA of 94.41% is the highest. The NB method achieves an accuracy of 84.65%. The precision of the SVM algorithm is 91.6%. Finally, RF provided an accuracy rate of 92.1%. The overall model performance is shown in Figure 8.

#### A. Sourced Data

**Figure 9:**
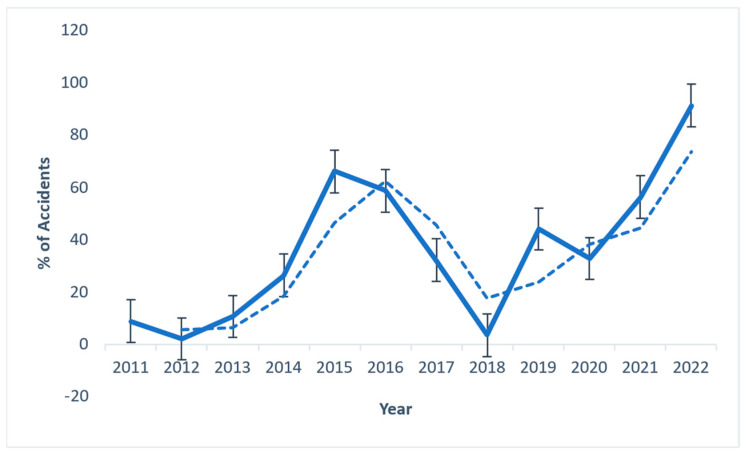
Numbers of accidents in the FI.

**Figure 10:**
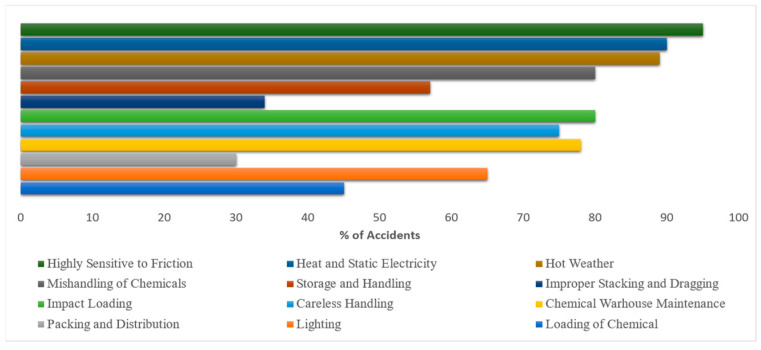
Number of accidents in each phase.

Accident data for the state’s FI came from the Chief Factory Inspectorate in Chennai, Tamil Nadu, and the Sivakasi Industrial Safety and Health Training Centre. The manufacturing process and the risks associated with the FI were reviewed during the fieldwork conducted for this research objective. The significant results were determined using a direct survey strategy. The Chief Inspectorate of Factories in Chennai and the Training Centre for Industrial Safety and Health in Sivakasi, Tamil Nadu, India, significantly contributed to the collection of accident datasets for the FI in Tamil Nadu, India. The data on accidents, which are displayed in Figure 9, make it abundantly clear that a significant volume of accidents occur yearly in the FI, and Figure 10 displays the proportion of accidents that occur in each step of the manufacturing process for the FI.

### 4.6. Expert System

ES is where AI has had the most significant impact on real-world applications. A knowledge-based system that can infer (reason) its way out of problems that would generally require HE is called an ES. ES are effective because of their extensive specialized knowledge about a single topic. ES is trained for customers to complement human decision makers, not as a replacement for them. ES does not capture him. They draw on their deep knowledge in a given field to solve problems and apply it to the available data. Heuristic knowledge, or “rules of thumb” used by HE in the domain, is also included in an ES’s knowledge base. At its fundamental, an ES consists of three primary components. There are three components: the inference engine, the knowledge base, and the user interface. An AI that can study HE and provide responses to challenging research questions.

*ES has the following advantages:*
*Result:* an improvement in the quality of decisions made.
*It is economical, reducing the cost and time* required to discuss with experts.
*Safe and effective services* to domain-specific problems.

XGBoost’s best-fit model is an ES. Rule-based ES solves the problem—this system’s primary input. Then, this work must write code in which the ML of the output (Y) depends on the inputs (X). To educate the computer to learn in this ES, give existing data and the corresponding output so that when a new input comes, it will predict the output and find the recommendation (Table 12).

All facilities that produce fireworks should have static discharge plates installed to prevent accidents because static electricity is hazardous in combustible or flammable atmospheres. Before an accident, workers can take preventive action by ranking the risks in various FI processes. Each process’s HE is identified, and safety measures are considered to reduce risk of death.

HE is reduced in different methods, including setting up a system, training employees, performing safety inspections, and encouraging open communication. Creating a system to reduce the potential for HE will help ensure that people do not make identical errors twice.

### 4.7. Recommendations to Prevent Accidents Caused by HE

Non-governmental organizations say the fireworks and match industries violate safety regulations, risking countless lives. The industry also hires unskilled children, continuing to increase accident rates. However, researchers propose the following safety precautions for industrialists preparing for the nation’s biggest festival.

(a) Automation of Hazardous Operations: Firecracker production can be automated. To reduce HE, machines can mix chemicals. The fireworks’ raw material is mixed and sieved many times. Accidents often result from work pressure. “Even manual processing is safe if workers are not pressured to produce more. Worker production targets should be realistic. The company follows factory and explosive safety rules. The FI can reduce accidents by using less sensitive raw materials.
(b) Factory-Based Manufacturing: Factory owners are increasingly hiring home workers. The hazardous industry now employs more children. Because it provides income for most of the population, some might even claim it is advantageous. “But it creates a process that turns children into full-time laborers”. Chemicals mishandled at home by non-manufacturers increase the risk of accidents.
(c) Pre-Employment Training: Untrained workers are given dangerous chemicals to mix and bind into explosive crackers. Accidents are more likely without training. Training is rare due to labor turnover. Workers switch jobs for even small pay increases. Industrialists do not want to invest in training workers who will leave soon.
(d) Stop the Cramming: In Sivakasi factories, four people share small cubicles. In the weeks leading up to Diwali, that number could double. High temperatures cause heat-reactive chemicals to explode. Lead poisoning, ulcers, and central nervous system damage can also result from prolonged chemical exposure in enclosed spaces. Sivakasi would see fewer deaths with better working conditions.

## 5. Conclusions and Future Work

This paper presents an expert system (ES) for the fireworks industry (FI) to reduce human errors (HE)-related accidents. This study analyzes the influence of HFs on the occurring HE. This analysis was implemented based on the Cross-Industry Standard Process for Data Mining methodology with machine learning (ML) and recommended the ES. The data collection and the questionnaire responses were reviewed for accuracy and coded. The Chief Inspectorate of Factories in Chennai and the Training Centre for Industrial Safety and Health in Sivakasi, Tamil Nadu, India, significantly contributed to the collection of accident datasets for the FI in Tamil Nadu, India. The database was created by entering the coded data into a computer. Data manipulation has been performed using the Statistical Package for Social Scientists (SPSS) software and MS-Excel Data Analysis. This study focused on the fireworks and match work industries in Tamil Nadu that were chosen to complete a questionnaire. Additionally, this research work was planned and performed to determine how much employees in the fireworks and match industries in Tamil Nadu interact with workplace safety and health data. The data were analyzed and presented in the following categories based on this study’s objectives: environmental, psychological, and organizational factors. ML identifies HE and safety measures to reduce risk of death. This research aims to create a new ES to mitigate HE risks in the FI. The proposed ES reduces HE risk and improves workplace safety in unsafe, uncertain workplaces.

In future work, the ensemble classifier technique will be used to combine different ML classifiers to achieve the best classification and prediction for the safety analysis of the FI. As a result, this research combines ML models and investigates other enhanced evolutionary algorithms to optimize other robust deep learning (DL) models to achieve higher accuracy in predicting firework safety management systems (SMS).

## Figures and Tables

**Table 1:** Summary of type of loss by multiple factors [61].

Type of Loss	Human Behavioral	Factors of Physical	External Financial Factors	Culture/ Environment
Direct Losses	Deaths and injuries Lost revenue Homeless people	Damage to the structure Contents damaged non-structurally Infrastructure Structural Failure	Business interruptions owing to building and infrastructure damage Response and relief capital costs	Depositional environment Emissions Vulnerable species Ecological zones are destroyed
Indirect Losses	Illnesses Disability Social disintegration	Damaged buildings and infrastructure deteriorate	Short-term activity disruption costs Permanent economic damage	Decreased biodiversity Reduced cultural types

**Table 2:** A breakdown of the percentages for personal factors and knowledge of workplace environment awareness.

Workplace Environments	V *	IV *	III *	II *	I *
Light Sources	267	69	11	67	7
Air Circulation	267	64	68	10	7
Fire Suppression System	324	26	50	10	7
Signs for Emergency Exits	87	28	50	190	64
Light for the Emergencies	25	52	11	10	321
Exits in Case of Emergency	290	28	11	10	80
Caution! Fire in the Building	41	13	50	72	243
Smoke Detection System	25	13	50	72	259
Storage of Flammable Liquids	25	67	237	67	23
Regular Electrical Maintenance	87	247	11	10	64
The State of Things in the Work Environments	267	28	107	10	7
Total = 4606	1705	635	656	528	1082
Percentage of Opinion	37.02	13.79	14.24	11.46	23.49

* V → Excellent IV → Very Good III → Good II → Satisfy I→ Not Satisfy.

**Table 3:** Analysis by percentage—effects of physical factors.

Descriptions	V *	IV *	III *	II *	I *
Headache	291	183	9	17	4.671
Tiredness	140	87	95	178	2.871
Weaknesses of the Human Body	127	175	198	0	3.742
High Blood Pressure	268	140	74	18	4.784
Spells of Heat or Cold	167	230	65	42	4.134
Heart Attack	147	86	57	210	3.456
Back Pain	110	266	87	37	4.134
Abnormality in the Throat	389	32	49	30	4.987
Ache in the Muscles	189	33	189	89	3.795
Quantitative Analysis of the Role of Physical Factors	3.656	2.464	1.646	1.242	0.0718

* V → Nobody Ever, IV → Rarely, III → Frequently, II → Daily, I → Weighted Average Score.

**Table 4:** Impact of physical factors on the test of the hypothesis.

Descriptions	Male	Female
Mean	27.89	22.11
SD	2.81	3.09

**Table 5:** Percentage analysis—impact of psychological workplace factors.

Descriptions	IV *	III *	II *	I *
Feeling Good	87	236	120	57
Feeling Tense	410	30	25	35
Anxiety Fear (Vague Fear)	389	45	54	12
Depression	389	54	56	1
Irritability	391	34	34	41
Mood Swings	399	89	5	7
Temper outbursts	387	52	16	45
Frustration	89	310	45	56
Boredom	290	34	98	78
Poor Concentration	314	49	46	91
Overall % of Psychological Factors	6.29	1.866	0.998	0.846

* IV → Never, III → Sometimes, II → Quite Often, I → Constantly.

**Table 6:** Verification of hypothesis—impact of psychological factors.

Description	Male	Female
Mean	46.19	53.81
SD	2.28	2.71

**Table 7:** Percentage analysis—impact of OF.

Descriptions	IV *	III *	II *	I *
Feelings of Being Alone	353	81	21	45
Prejudice	320	120	34	26
Untrustworthy	110	85	95	210
Decrease in Personal Connections	319	78	57	46
Emotions Isolated	317	67	27	89
Self Confidence	57	78	345	20
Lack of Confidence at Work	399	59	12	30
Overall Social Factors	46.88	14.20	14.78	11.65

* 4 → Never 3 Common → 2 Quite Often → 1 → Almost Constantly.

**Table 8:** Verification of hypothesis: the effects of OF.

Description	Male	Female
Mean	26.18	27.29
SD	2.81	2.15

**Table 9:** Rule-based classification analysis.

Rule-Based Classification	Actual Tag	
	HF	Non-HF
HF	82.5%	19.8%
Non-HF	17.3%	80.2%

**Table 10:** Performance of RFC.

Metric	Values
Accuracy	0.916
Precision	0.940
Recall	0.896
F1-score	0.921
AUC	0.890
Kappa Score	0.832
Error Rate	0.088

**Table 11:** Metrics of results for each model in the set.

Models	Accuracy	Precision	Sensitivity	Specificity	F1-Score
NB	86.45	90.5	79.8	74.5	84.5
SVM	91.6	86.1	96.2	60.0	87.1
RF	92.1	87.1	96.7	70.0	86.1
XGBoost	94.41	94.0	98.8	72.5	94.1

**Table 12:** ES recommendation (output).

EMP_AGE > 25	EMP_EXP > 10	EMP_TRAIN = Yes	EMP_STR = No	EMP_WH < 10	OUTPUT
1	1	1	1	1	1
0	1	1	1	0	1
1	0	1	1	1	1
1	1	0	0	0	0

## Data Availability

Not applicable.

## Abbreviations

Nomenclature		Abbreviations	
(x)	Transformations	AI	Artificial Intelligence
(X)	ML-Input	AUC	Area Under the RoC Curve
(Y)	ML-Output	BIA	Business Impact Analysis
$\sum_{J=1}^{J}$	Average Value of Tree Prediction	CA	Classification Accuracy
$\sum_{m=1}^{M}$	Efficiency and Speed	CDSS	Clinical-Diagnostic Decision Support Systems
$x_{i}.x_{j}$	Linear SVM	CRISP-DM	Cross-Industry Standard Process for Data Mining
‘k’	Specifies the Number of Groups	CSV	Comma-Separated Values
$\emptyset (x_{i}).\emptyset (x_{j})$	Non-Linear SVM	CV	Cross-Validation
c	Classes	DL	Deep Learning
C1	Probability of Placement	DT	Decision Trees
F	Class Name to Vote to Map	ES	Expert System
f(x)	Prediction Function	FCEM	Fire Cracker Explosives Manufacturer
F2(x)	Resulting Value of Prediction XGBoost	FI	Fireworks Industry
h1(x)	Prediction Value XGBoost	FIDMS	Fireworks Industry Disaster Mitigation System
k1 to k9	k-Fold CV	FN	False Negative
M	Tree Leaf Count	FP	False Positive
n	Pattern Space	GBM	Gradient Boosting
p	System’s Final Output	HF	Human factors
P(c)	Prior Probabilities for the class	IF	Influencing Factors
P(c|x)	Posterior Probability	IT	Information Technology
P(x)	Probability of the Predictor	KPI	Key Performance Indicator
q = f(p), p= (m, n)	AUC Value	ME	Misclassification Error
R	Set of Rules	MI	Mechanical Integrity
r	Rule	ML	Machine Learning
S	Worker’s Response	NB	Naïve Bayes
S′	Set of Sentences	OP	Operating Procedures
Si	Sentence List	PHA	Process Hazards Analysis
T	Path or Test Record	PSI	Process Safety Information
W	Class Name to Weight Mapping	QDA	Quadratic Discriminant Analysis
x	Exponential Function Value	RASH	Risk Assessment of Safety and Health
x, k	Feature Count Contribute	RF	Random Forest
σ	Sigma	SMS	Safety Management System
*X_i_*	Vectors	SVM	Support Vector Machine
$k(x_{i},x_{j})=exp[-\frac{\left|x_{i}-x_{j}\right|}{2\sigma^{2}}]$	Gaussian Radial Basis Function	TN	True Negative
$k(x_{i},x_{j})={\left(x_{i}.x_{j}\right)}^{d}$	Polynomial	TP	True Positive
$k(x_{i}.x_{j})$	Kernel Function	XGBoost	Extreme Gradient Boosting
		XLS/XLSX	Excel
